# Which support is provided in which country? Patterns among older adults in Europe

**DOI:** 10.1007/s10433-024-00808-y

**Published:** 2024-05-06

**Authors:** Emanuela Furfaro, Elvira Pelle, Giulia Rivellini, Susanna Zaccarin

**Affiliations:** 1 Department of Statistics, University of Washington, Seattle, WA USA; 2 Department of Communication and Economics, University of Modena and Reggio Emilia, Reggio Emilia, Italy; 3 Department of Statistical Science, Università Cattolica, Milan, Italy; 4 Department of Economics, Business, Mathematics and Statistics, University of Trieste, Trieste, Italy

**Keywords:** Social support, Ego-centered network, SHARE Wave 7, Country classification, MCA

## Abstract

This paper proposes comparative research on support provided outside the household by older adults in Europe. In studying social support, the network perspective is widely used, investigating, in particular, the ego-centered support networks of individuals. The analysis is based on data from Wave 7 of the Survey of Health Ageing and Retirement in Europe (SHARE). It examines the characteristics of ego-support networks of individuals aged 65 and over in 12 European countries, which are grouped into a novel welfare regime typology. Through Multiple Correspondence Analysis, we jointly looked into the categorical variables describing the recipients (alters) of the support provided by older adults and the welfare regime classification. As a main result, countries grouped in “*High degree of familialism by default*” category specialized in supporting family-related alters, particularly with childcare or personal care. In contrast, the elders in countries belonging to “*High degree of de-familialisation*” typology provided a more varied but less demanding support, to non-relatives alters and less oriented to care. The analyses show that the SHARE provides a solid opportunity to face the topic.

**Supplementary Information:**

The online version contains supplementary material available at 10.1007/s10433-024-00808-y.

## Introduction

Population aging has been a predominant phenomenon in twentieth-century Europe. It is a process driven by historically low fertility rates, increasing life expectancy, and, in some cases, migratory patterns (Eurostat 2020, 2022). According to the latest data published by Eurostat, the percentage of people aged 65+ years in the European Union -27 countries- was 21.1% on 1 January 2022, with an increase of 3 percentage points compared to 10 years earlier. Among these countries, Italy (23.8%), Finland (23.1%), Greece (22.7%), and Portugal (23.7%) had the highest shares, while Luxembourg (14.8%) and Ireland (15%) had the lowest, but constantly increasing shares. The process will most likely intensify throughout the current century (Grundy and Murphy 2017; Eurostat 2023; European Commission 2021). To deal with this process, societies need to promote active and healthy aging (Walker and Maltby 2012), a concept rapidly and broadly widespread in the scientific and political debate across Europe (Boudiny 2013; Zaidi and Howse 2017; Boerio et al. 2021). It is defined as the propensity to remain engaged in activities and community during the later stages of life (WHO 2002). Furthermore, in the analytical definition of the Active Ageing Index (UNECE/European Commission 2019), one of the domains addressed to grasp the concept of healthy and active aging is specifically devoted to participation in society which includes also social support.

Social support is viewed as a set of helpful functions exchanged between an individual and significant others, such as family members, friends, coworkers, relatives, and neighbors (Amati et al. 2017).

While support received by older adults has been largely studied, highlighting its positive influence on various health outcomes of the recipients, minor attention has been devoted to the support the elders provided to others (Fiori and Denckla 2012; Pelle et al. 2022). Providing support indicates an active lifestyle, usually associated with good mental and physical health. It can also contribute to maintaining “a sense of Purpose in Life”, recently underlined “in gerontological research as an important aspect of ageing well” (Bakhshandeh Bavarsad and Stephens 2024, p. 1). Specific types of help (e.g., personal or child care) are hardly given as a result of an individual choice free by family obligations and social norms (often gender-driven, e.g., see Kalmijn and Saraceno 2008; Schmid et al. 2012; Eggers et al. 2020) and the negative impact between caregiving and caregiver’s self-perceived health has been pointed out by many studies (for a review see Bom et al. 2019), particularly in the long term and for caregiving inside the household (Kaschowitz and Brandt 2017). However, a large body of literature has shown how being useful to others and still playing an active role in society can promote, especially for elders, a feeling of a worthwhile and meaningful life, enhancing either informal or formal social interactions (Litwin and Shiovitz-Ezra 2006; Rossi et al. 2014). The informal support provided by older adults is an important part of the overall care provision in many countries, also to compensate for the lack of services provided by governments and welfare systems to sustain both older adults (Pinquart and Sörensen 2003; Broese van Groenou and de Boer 2016; Bom et al. 2019; Kaschowitz and Brandt 2017) and younger generations (Attias-Donfut et al. 2005; Bordone et al. 2017; Glaser et al. 2010; Leitner 2003; Saraceno 2016; Zanasi et al. 2023). Consequently, in ageing societies, the role and the extent of older people in supporting others hold significant relevance at both the elder and the country levels.

In describing social support, the network perspective is widely used (Dykstra 2016; Amati et al. 2017; Furfaro et al. 2020; Lumino et al. 2016). In particular, it is often investigated through ego-centered support networks (Pelle et al. 2022; Perry et al. 2018), a specific type of network that maps an individual’s social connections.

Using data from the Survey of Health Ageing and Retirement in Europe (SHARE, Börsch-Supan et al. 2013) , we adopt an ego-centered perspective to analyze patterns of provided support among the elders in European countries. In particular, we focus on the support given to others outside the household thus providing a more grounded basis that offering support to individuals who do not share the same house daily can be less constrained by contingent circumstances and, likely, more related to an active lifestyle. Following the approach in (Lumino et al. 2016), using multidimensional analysis techniques, namely Multiple Correspondence Analysis (MCA, Greenacre and Blasius 2006) we synthesize the support provided, allowing cross-national comparisons and highlighting peculiarities in patterns of ego-centered support networks.

We contribute to the literature on support provided by older adults, by formulating and answering the following research questions which move from the hypothesis that patterns of support provided are different among European countries:

- *RQ1* What are common patterns of support provided outside the household by the elders in European countries?
- *RQ2* Does a country’s classification by welfare regime support the emerging patterns in providing social support to older adults?
- *RQ3* How does the data collection strategy in SHARE affect the definition and patterns of ego networks of support provided?

The rest of the paper is organized as follows: In the “Data and analytical approach” Section we provide information on the data including operational definitions of variables and the analytical approach adopted to answer our research questions. “Descriptive findings” Section presents the main descriptive findings on the observed ego-centered support networks, whereas the “Patterns of support through Multiple Correspondence Analysis of alters” Section is devoted to the analysis of the patterns of support provided which resulted from MCA. The “Discussion and concluding remarks” Section closes the paper.

## Data and analytical approach

### Data

We use data from Wave 7 of SHARE (Börsch-Supan 2019) with a focus on individuals aged 65+. From 2004, SHARE (https://share-eric.eu/) is a research infrastructure for studying the effects of health, social, economic, and environmental policies over the life course of individuals aged 50+ in 28 European countries and Israel.

The survey is organized into modules that focus on specific topics (e.g., health, social support, financial transactions, house conditions, etc.). In some of these modules, selected household members serve as family, financial, or household respondents who answer specific questions on behalf of the couple or of the whole household. In this paper, we consider Wave 7—carried out in 2017—since it is the last one completely carried out before the COVID-19 pandemic, which greatly impacted social interactions and consequently support.

Most of the information needed to build the support networks is contained in the Social Support module (SP). Thus, we work with individuals of age 65+ who responded to the SP module, yielding a sample of 11,390 individuals in 8,274 different households spread over the 12 countries (namely Austria, Belgium, Czech Republic, Denmark, France, Germany, Greece, Italy, Poland, Spain, Sweden, Switzerland).

### Using the SHARE SP module to build support networks

From the viewpoint of empirical measurement, the investigation of social networks, which describe the interaction and relationships among finite sets of individuals (Rafnsson et al. 2015), can be simplified by using the concept of an ego-centered network. This is defined starting from a focal individual (ego), who self-reports the set of other individuals (alters) around him/her (relatives, friends, neighbors, etc.) along with the type of relationship (emotional, informative, instrumental, etc) connecting them to ego through a “tie". When helpful functions (tangible or intangible) are exchanged between ego and significant others, then we can specify an ego-centered social support network (hereafter, only “support network"). Information on ego, such as age, gender, etc., is usually collected during the survey. Likewise, the type and frequency of support ties as well as demographic information on the alters may be available.

Using the SP module, we can investigate four types of support provided outside the household: personal care (1), practical household help (2), help with paperwork (3), whom we’ll refer to as *general support*, and *childcare* (4). As we tried to include general support and childcare in the same network, we encountered a challenge related to the data collection procedure. Some parts of the SP module are posed to all the individuals in the households, while others are posed solely to the family respondent, who answers on behalf of the couple or of the whole household. This results in missing values for those of the household who are not family respondents. Since we have no indications of how childcare responsibilities are shared within the couple/household (e.g. Di Gessa et al. 2020), we decided not to impute the missing values and to build support networks separately for the two groups of respondents and family respondents. The respondents’ support networks do not include childcare and the family respondents’ support networks which include general support and childcare.

#### Respondents’ support networks

**Fig. 1:**
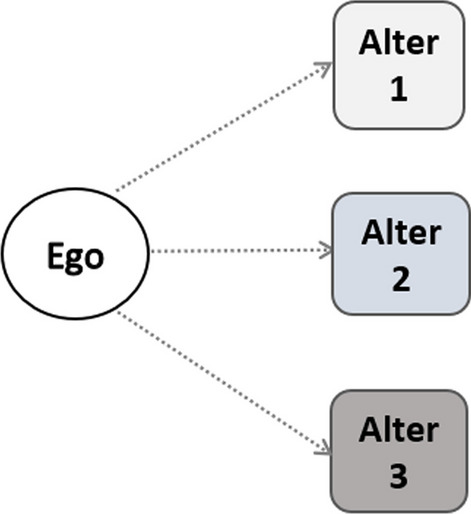
Respondents’ support network: possible provided support ties (dotted lines): (1) personal care, (2) household help, (3) help with paperwork. Alter *i*, ($i=1,2,3$): list of 28 role relations

The questionnaire item which investigates general support refers explicitly to support provided outside the household (“In the last 12 months, have you personally given any kind of help listed on this card to a family member from outside the household, a friend or neighbor?") and it allows the choice of up to three people from a list of 28 alter roles (e.g. Mother, Father, Child, Neighbor, Friend, etc.). If help is given, the respondent’s (ego) network size ranges from at least one alter to a maximum of three alters. Moreover, each alter can receive more than one type of support (multiple ties), as shown in Fig. 1.

From the question “In the last 12 months, how often altogether have you given such help to this person?" we can also derive the frequency of support provided to an alter.

#### Family respondents’ support networks

**Fig. 2:**
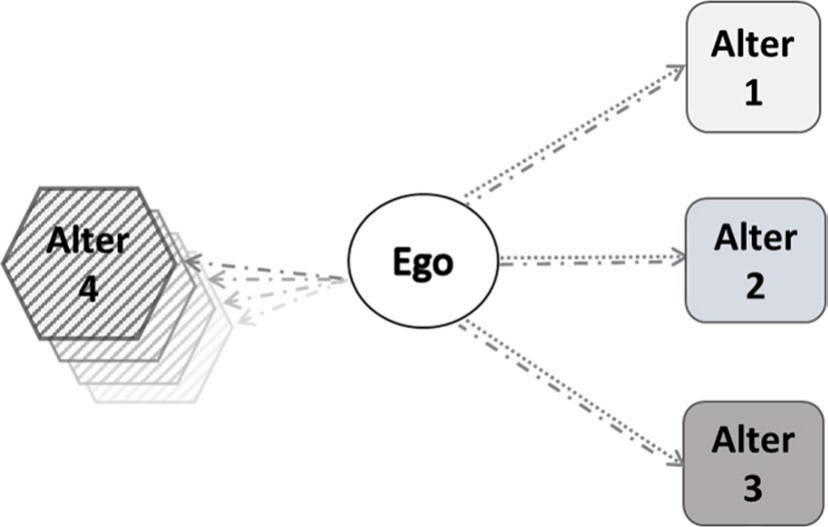
Family respondents’ support network: possible provided support ties: (1) personal care (dotted lines), (2) household help (dotted lines), (3) help with paperwork (dotted lines), (4) childcare (dash with dot lines). Alter *i*, ($i=1,2,3$): list of 28 role relations, *i* ($i \ge 4$): child

Childcare is investigated through a different questionnaire item devoted to family respondents only: “During the last 12 months, have you regularly or occasionally looked after your grandchild/your grandchildren without the presence of the parents?".^1^ A second question (“Which of your children [is the parent of the grandchild/are the parents of the grandchildren] you have looked after?") asks the family respondents to list all the children who benefited from their childcare. From this question, we can therefore derive up to as many alters as the number of children of the respondent, with all the resulting alters addressed in the “Child" role. The information derived from questions related to childcare was merged with the information on general support to create a network at the family-respondent level, as shown in Fig. 2. For this type of support and alter, the frequency of provided childcare is available from the survey.

### Socio-demographic variables and welfare regime typology

To provide a synthetic description of the elders (egos) providing support, we consider several socio-demographic characteristics (gender, geographical area,^2^ marital status, household type, number of children, age, education, residence area, health, health perceived, working status, having grandchildren), including the information on ’“caregiving” which is whether the respondent gave help to someone with personal care in the household (variable “caregiving”).^3^

As a first step towards answering RQ2, we propose a grouping of the countries into four categories of *welfare regime* that quite well overlaps both with the geographical areas above mentioned and with the UN AAI clustering (UNECE/European Commission 2019).^4^ In our proposal, we moved from the vast literature that stresses the importance of accounting for different institutions in the provision of welfare across Europe. Firstly the well-known welfare regime typology formulated by Esping-Andersen (1990) in his seminal work and further integrated with the crucial role of the family in the identification of a fourth regime that coincides with Mediterranean countries, the so-called familialistic one (Esping-Andersen 1999; Ferrera 1996). The subsequent and intense debate about the classification of countries based on the interplay between the family, the market, and the state has provided other useful insights for country classification. In particular, while some scholars have shown that Esping-Andersen’s typology still works when family-related issues are considered, others introduced new categorizations of countries that emphasize the role of specific areas of the provision of welfare. Albertini and Kohli (2013) have identified patterns of inter-generational support that overlap with Esping-Andersen’s typology of welfare regimes. These patterns are still very often mentioned in the interpretation of cross-national differences (Dykstra 2018; Leitner 2003; Saraceno and Keck 2010; Bordone et al. 2023). Conversely, a recent study (Bertin et al. 2021) showed that Esping-Andersen’s typology appears quite unstable and that a substantial revision is needed since the advent of the new Millennium. This is especially the case when scholars focus on certain areas of welfare provision. Taking into account healthcare and social care policies, they found a coexistence and an overlapping of multiple regimes - what is called a hybridization of welfare systems. This means that even in the same country, the welfare system is characterized by factors identified with more than one welfare regime. We then identified the Mediterranean regime (Italy, Spain, Greece with the inclusion of Poland) with the group of countries labeled *“High degree of familialism by default"*. Although highly heterogeneous, this first group is characterized by low state support of any form, in particular for the care of children aged under three. The families, rather than the state or the market, are considered responsible for the care and financial support of their dependent members, particularly young children and non-self-sufficient older adults. On the opposite side of the spectrum, we have Sweden and Denmark, which represent a group of countries with a *“High degree of de-familialisation"*. Namely, these countries are involved in a process aimed at lifting the burden of support from families through providing services offered either directly by the state or subsidized via the market (private services) (Floridi 2022). This group overlaps the ’social-democratic’ model of the Nordic countries. France and Belgium are grouped into a third group, which we called *“Towards a high degree of de-familialisation"*. Differently from the other Western and Eastern European countries, they perform well in the percentage of children under the age of three enrolled in formal care and even because the familialisation process is still optional (Floridi 2022). We then included Germany, Austria, Switzerland, and the Czech Republic into a residual group, simply named *“Other"*. While in the above-mentioned classification of Esping-Andersen, France, Belgium Germany, Austria, Switzerland are countries of one single model (the conservative one), this is not the case in our study. Nevertheless, we consider this new classification useful for understanding the patterns of support discovered in “Patterns of support through Multiple Correspondence Analysis of alters” Section.

### Multiple correspondence analysis

To obtain patterns of support and answer our research questions, we carry out a multivariate analysis of networks’ characteristics and welfare regimes. Since we deal with categorical variables, following (Lumino et al. 2016), we consider Multiple Correspondence Analysis (MCA, Greenacre and Blasius 2006; Everitt 2007) carried out on the alters as statistical units.

In MCA, categorical data are transformed into cross tables. Intuitively speaking, cross tables are useful to explore the associations between categorical variables in that they allow us to see which categories jointly appear more frequently thus indicating the existence of an association. When working with several categorical variables, the multidimensional cross-tables become much more difficult to interpret and multivariate analysis techniques that allow to synthesize the associations should be utilized. More technically speaking, given the contingency tables built on several categorical variables, the MCA extracts new dimensions that incorporate the original categories and reproduce the variability observed in the data set (Costa et al. 2013). By choosing a small number of dimensions—able to account for a large proportion of the observed variability, i.e. able to represent the original data set well—the data can be mapped into a lower dimensional space where each dimension represents several features jointly. These plots are called “factor maps" and allow us to map the patterns of support provided. The factor maps graphically represent the relationships between the original variables’ categories and their role in defining the dimensions represented on the *X* and *Y* axes. Variable categories that are positively associated are grouped on the plot, whereas negatively associated variable categories are positioned on opposite sides of the plot origin. By observing such plots we can see how the categories contribute to the new dimensions and we let patterns of support emerge.

Therefore, we use MCA and the factor maps to examine patterns of support emerging from the characteristics of the alters in the support networks. This helps us answer RQ1 and RQ2.

## Descriptive findings

### Support providers

Table 1 reports the number of respondents per country grouped into the four welfare regime categories, along with the percentage of support providers in the sample (unweighted data). These figures are presented separately for all respondents and family respondents. Among the 11,390 individuals aged 65+ who undertook the SP module, a subgroup of $n_{\textrm{Resp}} = 2629$ (23.1%) provided general support, while for the 8274 family respondents, the percentage of support providers ($n_{\textrm{FamResp}} = 3514$) is 42.5%. In particular, 30.7% of family respondents providing support, offered only general support, 44.2% only childcare, and 25.2% provided both types of support. In Denmark, older adults appear highly committed to providing support with nearly one over two being engaged in providing support outside their household, whereas in Spain, Italy, and Greece, older adults seem to be less active. As we extend the definition to include childcare for family respondents (see two right-most columns of Table 1), Denmark and Sweden keep experiencing the highest percentages of support providers, while Italy, Spain, and Greece continue to show low engagement.

**Table 1:** Respondents or family respondents who provided support outside their household in the last 12 months (%, unweighted data)

Welfare regime typology	Country	Respondents (general support)		Family respondents				
		*n*	Support providers	*n*	Support providers	Only general	Only childcare	Both
High degree of de-familialisation	Denmark	952	47.5	715	60.7	37.8	23.7	38.5
	Sweden	980	32.9	761	54.7	23.1	38.7	38.2
Towards a high degree of de-familialisation	Belgium	1289	26.2	945	47.9	30.5	45.5	24.1
	France	894	32.1	669	51.9	32.9	37.8	29.4
High degree of familialism by default	Greece	1458	8.6	1058	21.2	26.8	61.6	11.6
	Italy	1335	11.2	906	36.4	16.7	67.0	16.4
	Poland	874	11.3	594	37.0	20.5	68.2	11.4
	Spain	1084	7.3	764	29.5	14.7	74.7	10.7
Other	Austria	435	27.6	338	38.2	46.5	28.7	24.8
	Czech Republic	775	28.1	548	46.5	38.8	36.1	25.1
	Germany	699	32.9	503	49.7	43.2	31.6	25.2
	Switzerland	615	34.0	473	48.6	45.7	28.7	25.7
	Total	11,390	23.1	8274	42.5	30.7	44.2	25.2

Own elaborations on SHARE Wave 7 data.

**Fig. 3:**
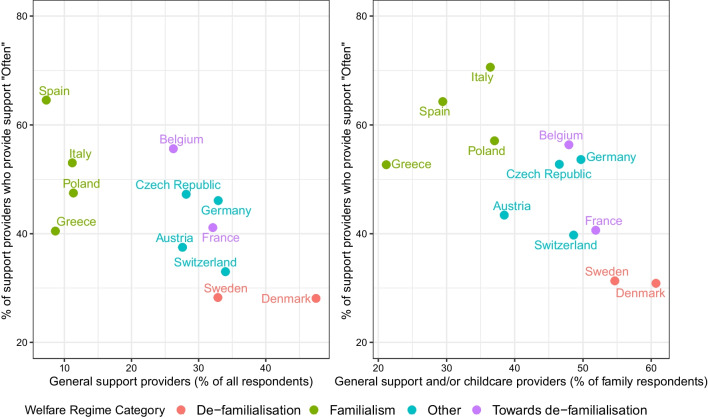
Support providers (%) versus support provided “Often" to alter 1 (%) among respondents (left panel) and family respondents (right panel)

However, reading this result along with the frequency of support provided, we notice that countries characterized by a “*High degree of de-familialisation*” show the lowest engagement in terms of frequency, while Italy, Greece, Spain, and Poland, are characterized by the highest involvement in terms of frequency of providing support. This trend is made explicit in the scatter plots of Fig. 3 where countries with higher percentages of support providers also exhibit lower percentages of frequent support (Frequency=“*Often*").

To highlight potential differences between support providers and the respective whole samples, Table 2 shows the socio-demographic characteristics of the corresponding groups. The conditional distributions of support providers with respect to the marginal distributions of the two groups (respondents and family respondents) are statistically different according to the $\chi ^2$ test for almost all the considered characteristics^5^.

**Table 2:** Support providers by socio-demographic characteristics

	Respondents		Family respondents	
	All	Support providers	All	Support providers
	(*n* = 11,390)	(*n* = 2629)	(*n* = 8274)	(*n* = 3514)
*Gender*				
Male	44.4	46.9	40.3	41.5
Female	55.6	53.1	59.7	58.5
*Geographical area*				
Northern Europe	17.0	29.4	17.8	24.2
Western Europe	34.5	45.0	35.4	40.1
Southern Europe	34.0	13.5	33.0	22.2
Eastern Europe	14.5	12.1	13.8	13.5
*Welfare regime typology*				
High degree of de-familialisation	17.0	40.1	17.8	57.6
Towards a high degree of de-familialisation	19.2	28.6	19.5	49.6
High degree of familialism by default	41.7	9.5	40.1	30.1
Other	22.2	30.8	22.5	46.5
*Marital status*				
Married	65.9	65.7	52.6	61.3
Widowed/divorced	28.2	26.6	38.4	32.1
Other	5.9	7.7	9.0	6.6
*Household type*				
Couple	53.9	59.3	42.8	53.1
Single	29.6	29.9	40.7	33.2
Other	16.5	10.9	16.5	13.7
*N. of children*				
0	9.6	8.0	11.4	5.4
1	16.4	16.9	16.6	15.7
2	41.2	43.2	39.5	43.6
3 and more	32.8	31.8	32.4	35.3
*Age*				
65–69	27.0	38.2	25.5	36.3
70–79	45.3	49.2	43.8	50.8
80+	27.7	12.6	30.6	12.9
*Education*				
Low	40.7	29.7	43.7	35.3
Medium	34.4	38.0	31.2	36.5
High	19.9	29.8	19.4	25.4
Other	4.9	2.4	5.7	2.7
*Residence area*				
Big city	14.2	11.8	14.7	12.7
Suburbs^a^	10.0	12.6	10.1	11.4
Large town	17.3	16.5	17.5	16.7
Small town	23.3	23.5	23.1	23.8
Rural area/village	32.2	32.9	31.3	32.6
Missing^b^	3.0	2.7	3.2	2.9
*Health-Gali*				
Limited	50.0	42.1	50.6	42.1
Not limited	50.0	57.9	49.3	57.9
*Health perceived*				
Poor^c^	41.8	27.6	41.9	30.1
Good^d^	58.2	72.4	58.1	69.9
*Working status*				
Retired	83.7	87.1	81.8	85.4
Employed^e^	3.5	5.2	3.5	4.4
Homemaker	9.5	4.9	9.0	7.1
Other	3.3	2.8	5.7	3.2
*Grandchildren*				
Yes	82.1	80.3	78.3	80.5
No	17.9	19.7	21.7	19.5
*Caregiving*				
Yes	7.2	7.5	6.6	6.6
Not	66.1	64.9	56.9	62.7
Not applicable	26.7	27.7	36.4	30.8

^a^Includes “Suburbs” and “Outskirts of the big city”

^b^Includes “Refusal” and “Don’t know”

^c^Includes “Fair" and “Poor"

^d^Includes “Good", “Very good" and “Excellent”

^e^Includes “Employed" and “Self-employed"

### Support networks

We now describe the observed support networks by countries classified by the welfare regime categories, focusing on size and network typology. Size is obtained as the sum of the number of alters declared, while the typology is obtained by aggregating the 28 role relations - listed both for general and childcare support - into four categories.^6^ Tables 3 and 4 report respectively the size and the type of the networks of provided general support for respondents, while Tables 5 and 6 show the same two characteristics for the networks of provided general support and/or childcare for family respondents. We offer a slight modification of the network types proposed in the literature (Litwin et al. 2020; Furfaro et al. 2020), which usually distinguish between networks with family alters, non-family alters, and a mixed type of alters. In particular, we add the typology “Offspring” in addition to the more traditional “Family”, “Non-family”, and “Mixed” to better represent the variability in network types/alters observed among countries.

Table 3 highlights that most of the networks are fairly small, containing mainly only one alter. Denmark, Belgium, and Austria exhibit the highest percentages of two or three alters in the support networks, while Greece and Spain exhibit the smallest ones. This confirms the preliminary observations in Table 1 on engagement in support outside household by countries. More specifically, the mean values by welfare regime typology show a clear difference between the countries belonging to the category “*High degree of familialism by default*” and the other.

Table 4 shows a certain variability in types among countries. Nevertheless, family-only networks are predominant in countries such as Greece, Italy, and Spain, still belonging to the category “*High degree of familialism by default*”. Countries with "Non-family" networks as their predominant typology are instead Switzerland, Sweden, Germany, and Denmark not so clearly distinguished by welfare regime typology.

**Table 3:** Respondents’ support networks’ size by welfare regime typology and country (general support) %

Welfare regime typology	Country	Number of alters		
		1	2	3
High degree of de-familialisation	Denmark	63.9	21.2	14.8
	Sweden	70.5	19.6	9.9﻿
	Mean (%)	66.7	20.5	12.8
Towards a high degree of de-familialisation	Belgium	64.5	24.3	11.2
	France	72.8	19.2	8.0
	Mean (%)	68.3	21.9	9.8
High degree of familialism by default	Greece	87.2	11.2	1.6
	Italy	78.5	17.4	4.0
	Poland	82.8	16.2	1.0
	Spain	86.1	10.1	3.8
	Mean (%)	83.2	14.2	2.7
Other	Austria	68.3	19.2	12.5
	Czech Republic	70.6	18.3	11.0
	Germany	70.0	24.3	5.7
	Switzerland	77.0	16.3	6.7
	Mean (%)	71.8	19.7	8.5

Own elaborations on SHARE Wave 7 data.

**Table 4:** Respondents’ support networks’ types by welfare regime typology and country (general support) %

Welfare regime typology	Country	Network type			
		Family	Mixed	Non-family	Offspring
High degree of de-familialisation	Denmark	21.9	14.2	36.1	27.9
	Sweden	23.4	9.3	39.3	28.0
	Mean	22.5	12.2	37.4	27.9
Towards a high degree of de-familialisation	Belgium	31.4	13.6	31.1	24.0
	France	27.2	15.0	37.3	20.6
	Mean	29.4	14.2	33.9	22.4
High degree of familialism by default	Greece	52.0	idem	18.4	25.6
	Italy	42.3	6.0	31.5	20.1
	Poland	36.4	4.0	40.4	19.2
	Spain	41.8	5.1	34.2	19.0
	Mean	43.6	idem	30.3	21.2
Other	Austria	20.0	19.2	31.7	29.2
	Czech Republic	23.4	13.3	28.0	35.3
	Germany	22.6	10.4	39.6	27.4
	Switzerland	21.1	9.6	49.3	20.1
	Mean	22.0	12.4	37.7	27.9
	Total	27.6	11.5	35.4	25.5

Own elaborations on SHARE Wave 7 data.

The network size of family respondents is larger (Table 5) since we allow the presence of more alters. In Greece, Italy, and Poland most of the sampled elders provide support to only one alter, in Sweden and Denmark the proportion of networks with more than one alter now represents nearly half of the networks. The network types, represented in Table 6, exhibit heterogeneity across countries. The predominant network type in Spain, Italy, Poland, and Greece, is "Offspring", with very little room for any other alter type. On the other hand, Denmark, Austria, Switzerland, and France exhibit more heterogeneity with a significant presence of networks with "Mixed" or "Non-family" alters. The mean values for “*High degree of de-familialisation*” are now much higher in comparison to the other three categorizations.

**Table 5:** Family respondents’ support networks’ size by welfare regime typology and country (general support and/or childcare) %

Welfare regime typology	Country	1	2	3	4+
High degree of de-familialisation	Denmark	47.7	28.8	19.6	3.9
	Sweden	48.1	34.1	13.9	3.8
	Mean	47.9	31.4	16.8	3.9
Towards a high degree of de-familialisation	Belgium	54.5	27.8	13.5	4.2
	France	53.0	30.3	13.5	3.2
	Mean	53.9	28.9	13.5	3.8
High degree of familialism by default	Greece	77.2	18.3	4.5	0.0
	Italy	65.2	27.0	7.0	0.9
	Poland	72.7	20.9	5.0	1.4
	Spain	65.3	23.6	8.0	3.1
	Mean	69.6	22.9	6.2	1.3
Other	Austria	63.6	23.3	11.6	1.6
	Czech Republic	61.2	25.5	10.6	2.7
	Germany	60.0	27.2	10.8	2.0
	Switzerland	60.9	23.5	12.2	3.5
	Mean	61.1	25.1	11.2	2.5

Own elaborations on SHARE Wave 7 data.

**Table 6:** Family respondents’ support networks’ types by welfare regime typology and country (general support and/or childcare) %

Welfare regime typology	Country	Network type			
		Family	Mixed	Non-family	Offspring
High degree of de-familialisation	Denmark	16.8	21.4	17.7	44.0
	Sweden	14.4	17.5	12.7	55.3
	Mean	15.6	19.5	15.3	49.5
Towards a high degree of de-familialisation	Belgium	16.1	12.6	13.7	57.6
	France	17.3	17.9	14.7	50.1
	Mean	16.6	14.9	14.1	54.4
High degree of familialism by default	Greece	18.3	3.1	6.3	72.3
	Italy	12.7	7.9	5.8	73.6
	Poland	idem	5.9	9.5	75.0
	Spain	10.7	4.0	5.3	80.0
	Mean	12.8	5.5	6.6	75.1
Other	Austria	15.5	17.8	18.6	48.1
	Czech Republic	13.7	13.7	13.3	59.2
	Germany	14.0	12.8	24.0	49.2
	Switzerland	16.1	17.0	24.8	42.2
	Mean	14.7	14.9	20.3	50.1
	Total	14.8	13.4	13.8	58.0

Own elaborations on SHARE Wave 7 data.

**Fig. 4:**
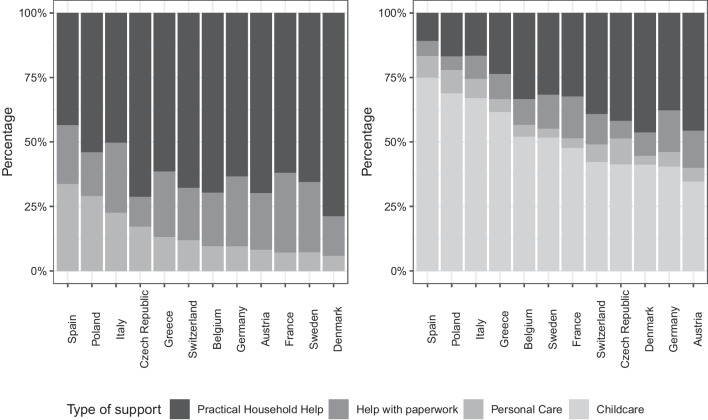
Bar-plots of type of support provided in respondents’ support networks (left panel) and family respondents’ support networks (right panel) by country

Lastly, the plots in Fig. 4 represent the type of support provided to each alter in the respondents’ and the family respondents’ networks. From the left panel, we can see that in countries with “*High degree of familialism by default*" (Italy, Spain, Poland, Greece), there is a higher percentage of “Personal Care" as a type of support. We can interpret this type of support as one provided to people who are not self-sufficient and therefore it is reasonable that this is more common in countries characterized by low state support. In the family respondents’ networks (right panel), we see that it is again more prevalent in the same countries. Personal care and childcare together make up over 75% of the type of support in familistic countries, while they represent a smaller proportion in the “*High degree of de-familialisation*” countries.

## Patterns of support through Multiple Correspondence Analysis of alters

The previous analysis showed that the differences between countries grouped into welfare regime categories involve several characteristics of the support networks, including the frequency of support provided, the number of alters, the alter type, and the type of support. These are factors that can contribute to the definition of patterns of support, and they can be more efficiently treated if jointly considered. As mentioned in “Multiple Correspondence Analysis” Section, following (Lumino et al. 2016) we use MCA and in particular we consider the alters as statistical units. In our application, the first two dimensions extracted by the MCA accounted for a large proportion of the total variability both in the respondent network and in the family respondent network. The subsequent dimensions did not add significant value to the analysis, neither in terms of additional variability explained nor in terms of interpretation of patterns of support. Therefore, we deemed the first two dimensions enough to provide a picture of the most evident types of support. More detailed results on the proportion of variability explained can be found in the Appendix (Additional file 1).

**Fig. 5:**
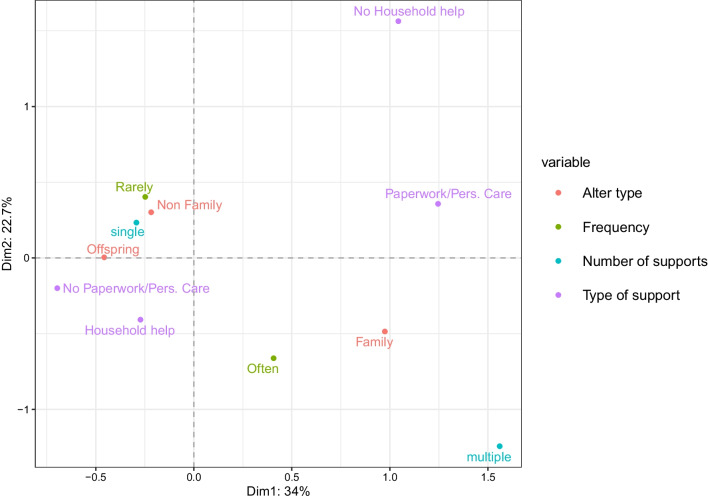
Factor map of the considered variables for the respondents’ support networks. Variable names in legend. Variance explained by dimension 1 on the *x*-axis: 34%. Variance explained by dimension 2 on the *y*-axis: 22.7%

First, we look at the respondents’ support network, where the first two dimensions account for 57.1% of the total variability. In Fig. 5, the first dimension (*X*-axis) polarizes the patterns of support. On the right-hand side, the category of “Family" alters is in the same quadrant as multiple support and personal care/paperwork types of help categories. On the left-hand side, “Household help" - provided, although rarely, as the only type of support (single) - is closer to “Offspring". From this visualization, two main patterns of support can be recognized. The first one is characterized by personal care activities towards close relatives (i.e., siblings), probably in the same age range as ego. The second one is related to a more specific and practical activity offered occasionally to their children and probably not very demanding for the elders. Both patterns primarily focus on alters tied to the elders by family relations. With regards to non-family members, the results show a sporadic type of support, with the categories “Non-Family", “Rarely" and “Single" being all clustered together, suggesting a lower-involvement pattern in providing support to alters who are not in the kinship sphere.

**Fig. 6:**
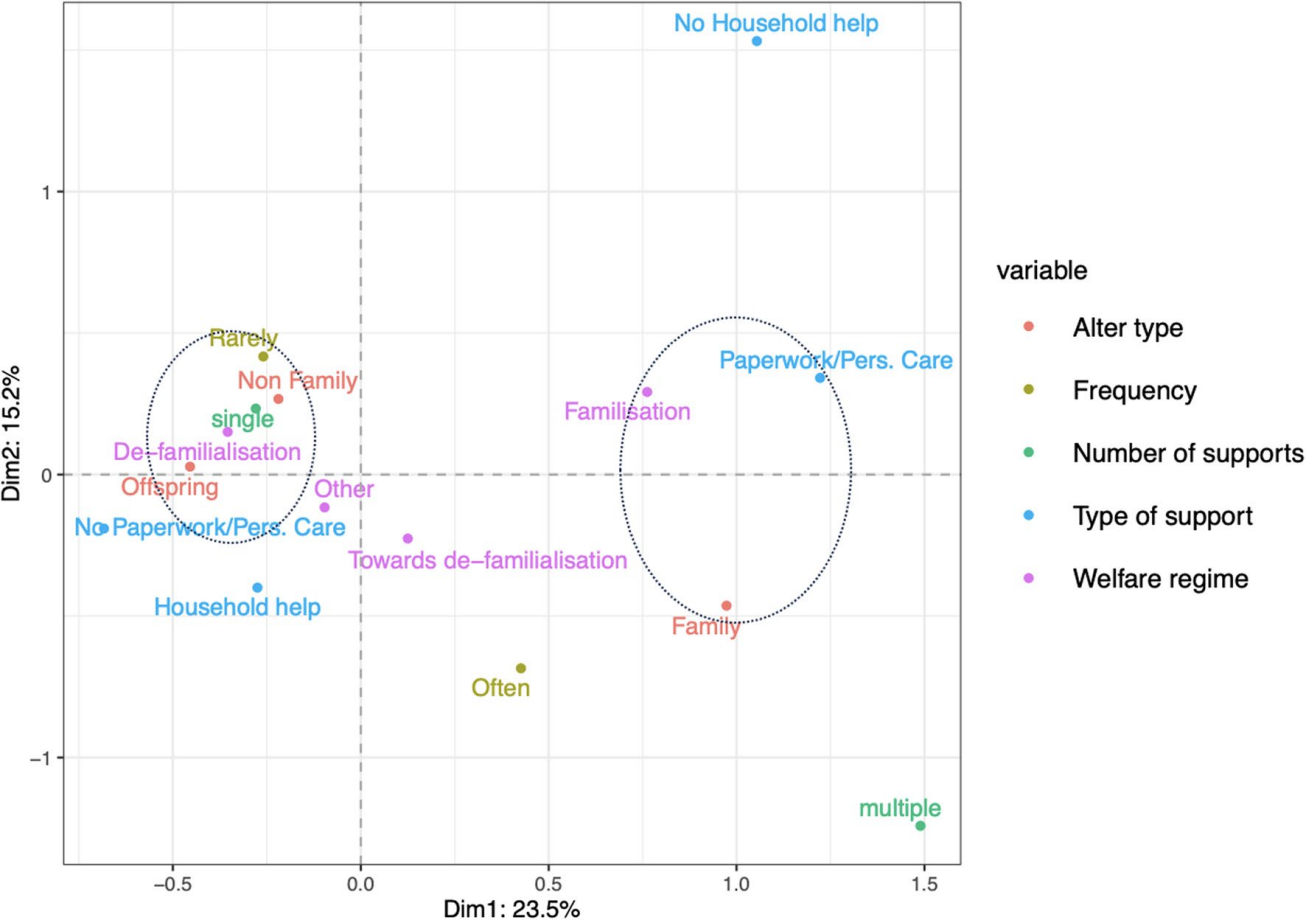
Factor map of the considered variables, including welfare regime typologies, for the respondent’s support networks. Variance explained by dimension 1 on the *x*-axis: 23.5%. Variance explained by dimension 2 on the *y*-axis: 15.2%

We now consider whether these patterns are more or less typical of specific groups of countries. Figure 6 shows that the right-hand side pattern is associated with a welfare regime strictly familistic, while the pattern observed to the left is more typical of Northern European countries, characterised by non-familistic welfare systems. The two remaining categories of welfare typologies do not seem to be particularly associated with any of these two patterns.

**Fig. 7:**
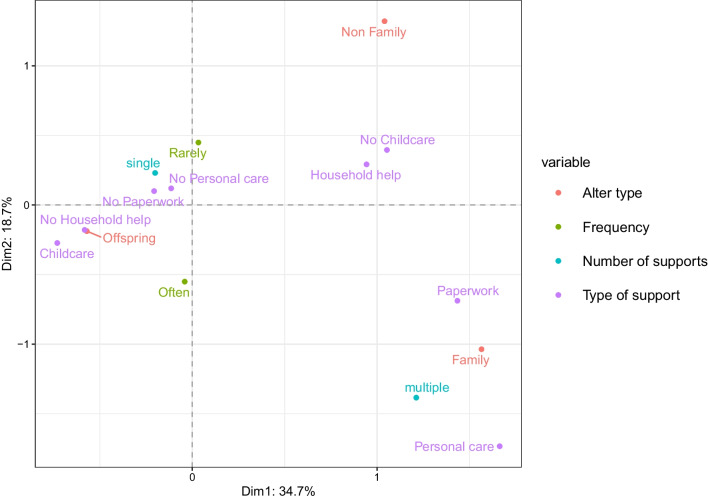
Factor map of the considered variables for the family respondents’ support networks. Variance explained by dimension 1 on the *x*-axis: 34.7%. Variance explained by dimension 2 on the *y*-axis: 18.7%

**Fig. 8:**
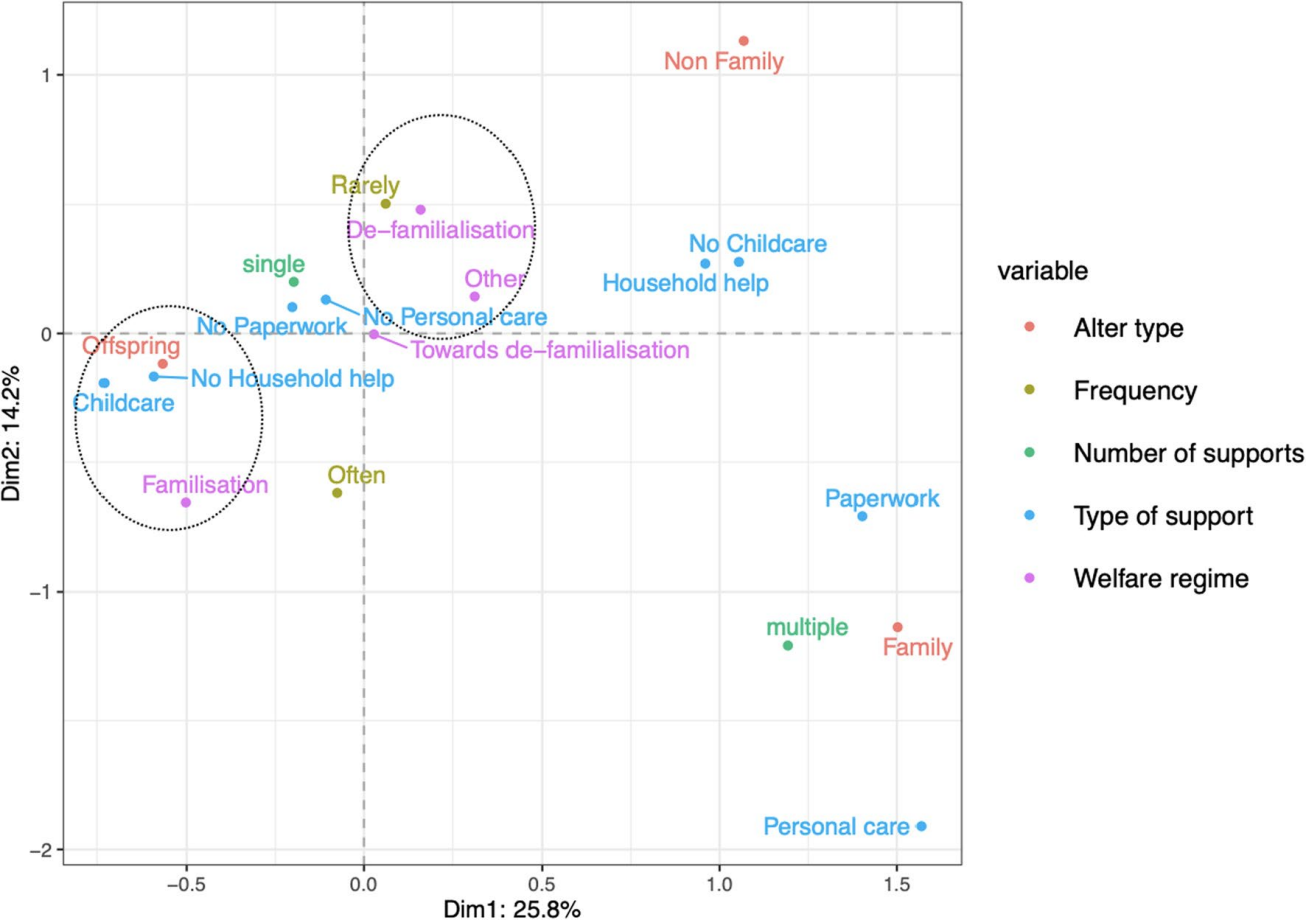
Factor map of the considered variables, including welfare regime categories, for the family respondents’ support networks. Variance explained by dimension 1 on the *x*-axis: 14.2%. Variance explained by dimension 2 on the *y*-axis: 25.8%

Regarding the family respondents’ support networks, which include childcare, Fig. 7 shows that the two dimensions divide the plot into two main areas, and a third less evident one, suggesting two main patterns of support. On the left-hand side, childcare is the predominant type of support and it is provided, by definition, exclusively to their children. In the bottom right corner, we find a pattern similar to the one described in Fig. 5, with family members (generally parents or siblings of ego) receiving multiple supports, personal care, and help with paperwork. In the top right corner, we see that support provided to non-family alters is separate and does not tend to correspond to any of the two described patterns. As we again add the welfare regime categories, in Fig. 8 we can see that the left-hand side pattern of support encapsulated in the family circle and provided multiple times per week or daily is associated with countries where the family is still a crucial institution. Less defined appear the patterns on the right-hand side of the *X*-axis, while along the *Y*-axis we can detect a pattern of support provided rarely for countries involved in the de-familialisation process.

## Discussion and concluding remarks

In this paper, we used SHARE data to study support provided by the elders in Europe. The data enabled us to investigate individuals’ supportive relationships, examining changes across countries in terms of frequency, type of support, and the number and type of recipients (alters). Moving from the hypothesis that patterns of support provided outside the household are different among European countries, we jointly analysed network data and welfare regime typologies through MCA.

Our analysis allows us to identify some common patterns of support provided among European countries (RQ1). When looking at the networks built without childcare, two main patterns of support can be recognized. The first one is characterized by personal care activities towards close relatives, and the second one regards household help occasionally provided to children. When shifting on the family respondents’ support networks, which include childcare, we obtain two main patterns, one which highlights childcare and one which highlights other types of support provided to family members, and a third, less evident, which summarises help to non-family alters.

To answer RQ2, we read the results with the welfare regimes classification. In doing so, we highlighted differences between two of the proposed welfare regime categories, with countries grouped in the “*High degree of familialism by default*” typology specializing in support to family-related alters, particularly childcare or personal care. We expect the support provided to relatives outside the household to be care-oriented, either towards children, the disabled, or other older adults. Further, in this typology, females are mainly involved in this type of support, while males are more involved in providing types of general support^7^. This pattern is in line with the findings in (Bordone and Arpino 2018), which identified three groups of social participation among older adults, one of which is characterized by a high concentration of care activities. Other countries (grouped into a category that recalls the ‘social-democratic’ model of the Nordic countries) offer more varied but less demanding support, non-family-related alters, and less oriented to care. This seems reasonable since the goal of the “de-familialisation" process is to lift the burden of support from families through the provision of services offered either directly by the state or subsidized or provided via the market (Floridi 2022). This different specialization could explain a more limited involvement of elders in other forms of participation in society. Mediterranean countries (with the inclusion of Poland) show indeed the lowest overall AAI scores, while Sweden and Denmark present well above average results in all domains of the AAI. We are aware that the welfare regime classification we propose does not lead to a clear identification of common patterns for countries in continental Europe, for which more fuzzy results emerge. Similarly, with this new categorization, it becomes more difficult to address differences between Eastern and Western countries. First, because in the new classification, the “*Other*” category includes both Western and Eastern countries. Then, this category and the “*Towards a high degree of de-familialisation*” (with only Western European countries inside) as well were not so clearly detected in MCA as the categories “*High degree of de-familialisation*” and “*High degree of familialism by default*”. Further, the only two Eastern European countries considered in the analysis (Poland and Czech Republic) behave quite differently both with respect to the observed support networks and the AAI score. Poland fits well with the “*High degree of familialisation*” category, while the Czech Republic with the more nuanced category “*Towards a high degree of de-familialisation*”. Poland is therefore inserted by the UN in the cluster with the lowest overall AAI index, while the Czech Republic exhibits an intermediate score. The association of the above main patterns with the welfare regime typology appears only for some categories, thus providing only a partial answer to RQ2. In particular, for Central and Eastern European (CEE) countries additional research is needed in order to better grasp the complexity of the recent transformation of the welfare system and its dynamic reaction to changing social needs. The results we presented can be a starting point for future analysis of similar topics focused on CEE countries. SHARE can offer this opportunity since new countries are involved in the next waves (i.e. Estonia, Hungary, Latvia, Lithuania, Romania, Slovak Republic, and Slovenia).

With regards to our last research question (RQ3) we also tried to handle the often-overlooked problem of working on SHARE items answered by different respondents within the same household. While a large body of literature focused on grandparenting and childcare as the main supportive activity provided by elders (Bordone et al. 2017; Floridi 2022; Glaser et al. 2010; Jennings et al. 2021; Zanasi et al. 2023), in this paper we aimed to consider various types of support to make the networks as comprehensive as possible thus offering a complete view of the involvement in providing support by elderly people. In the data collection strategy used in SHARE, questions related to childcare are answered by family respondents who respond on behalf of the couple. Since providing care for grandchildren may impact grandmothers and grandfathers differently, it is difficult to make assumptions about how childcare responsibilities are shared by the couple. Moreover, the family respondent could answer not strictly at a personal level, because he/she could think more about the couple/household and not him/herself. Researchers either impute the value to the respondent’s partner or they work only on the family respondents, limiting flexibility in defining and answering research questions and limiting the use of a univocal and shared approach. Therefore, we decided to build two different types of support networks: the respondents’ networks which include general support provided at the individual level, and the family respondents’ networks with information on grand-parenting.

The value added by working separately on the networks defined on all respondents and family respondents only is twofold: it allows us to produce knowledge on the support provided by all types of respondents and it allows us to have results on a larger sample. While researchers interested in childcare will likely work on family respondents, researchers interested more in general support will work on all the respondents. Since SHARE is one of the largest and most valuable data source on aging, the issue of support data collection strategy should be addressed. Similarly, more detailed information on personal care activities performed, especially in terms of the frequency with which these activities are conducted, could help further clarify the role of the elders as supporters in aging societies. One of the strengths of using SHARE data carried out since 2004 is in making our research replicable. Future research could be devoted to the expansion of this topic to other waves of SHARE and to the study of the dynamics of provided support networks with the aim of exploring how and if patterns have changed throughout the pandemic. In addition, the definition of networks of provided support given in this paper can be used to carry out a longitudinal analysis of support networks.

### Supplementary Information

**Additional file 1. Appendix:** MCA details.

## Footnotes

1 This question does not explicitly refer to grandchild/grandchildren outside the household. Since in our dataset (unweighted data), the percentage of people who live with their children and grandchildren is negligible (roughly 1%), we can refer to childcare as a type of support provided to non-cohabiting children.

2 Aggregated in Northern Europe (Sweden, Denmark), Western Europe (France, Switzerland, Belgium, Germany, Austria), Southern Europe (Spain, Italy, Greece), Eastern Europe (Czech Republic, Poland).

3 The specific question is the SP018_GiveHelpInHH: Let us now talk about help within your household. Is there someone living in this household whom you have helped regularly during the last 12 months with personal care, such as washing, getting out of bed, or dressing?

4 In UNECE/European Commission (2019), Sweden and Denmark have been grouped in the “Nordic” cluster, which presents well above average results in all domains of the AAI. Italy, Spain, Poland, and Greece are in the cluster with the lowest overall AAI score. France, Belgium, Germany, Austria, and the Czech Republic exhibit intermediate AAI scores.

5 *p* values were $< 0.001$, except the “gender’ and “number of children” in the respondent group with *p* values $< 0.003$.

6 “Family" (1), composed only by alters from immediate family, close and other relatives; “Offspring" (2) of ego and their descendants (child, step-child/current partner’s child, son/daughter-in-law, grandchild); “Non-family" (3) composed only by neighbors, friends, co-workers or others; “Mixed" (4) by at least one alter from Family/Offspring and Non-family types.

7 For the typology "*High degree of familialism by default*” the percentage of females providing general support is 59% and general and/or childcare is 62%. The highest percentage of males providing general support (50%) is observed for the "*High degree of de-familialisation".*
