# Supplementary material for: Which support is provided in which country? Patterns among older adults in Europe
Source: Eur J Ageing. 2024 May 6;21(1):15. doi: 10.1007/s10433-024-00808-y (PMC11070411; doi:10.1007/s10433-024-00808-y)
Supplement: Supplementary file 1 — Additional file 1. Appendix: MCA details. [file 10433_2024_808_MOESM1_ESM.pdf]

Which support is provided in which country? Patterns among older adults in Europe

European Journal of Ageing

Appendix: MCA details

Variable	Category	Dim 1	Dim 2	Dim 3	Dim 4	Dim 5
Type of alter	Family	0.97	-0.49	0.14	-0.44	-1.25
	Non Family	-0.22	0.30	-1.10	0.23	0.31
	Offspring	-0.46	0.00	1.17	0.05	0.55
Multiple support	Multiple	1.56	-1.24	-0.23	-0.68	0.90
	Single	-0.29	0.23	0.04	0.13	-0.17
Frequency of support	Often	0.41	-0.66	0.06	1.02	-0.06
	Rarely	-0.25	0.40	-0.04	-0.62	0.03
Type of support	Household help	-0.27	-0.41	-0.07	-0.11	0.04
	No Household help	1.04	1.56	0.27	0.44	-0.16
	Paperwork/Pers. Care	1.25	0.36	0.05	-0.05	0.31
	No Paperwork/Pers. Care	-0.70	-0.20	-0.03	0.03	-0.17

Table 1: Coordinates of variable categories on the first 5 dimensions for values displayed in Figure 5. Columns Dim 1 and Dim 2 were used as the X and Y axis in Figure 5.

Dimension	Var. Explained	Cum. Var. Explained
dim 1	33.99	33.99
dim 2	22.69	56.68
dim 3	16.58	73.26
dim 4	13.96	87.22
dim 5	12.59	99.81
dim 6	0.19	100.00

Table 2: Variability explained by the dimensions extracted in MCA (%) reported in Figure 5.

Variable	Category	Dim 1	Dim 2	Dim 3	Dim 4	Dim 5
Type of alter	Family	0.97	-0.46	0.36	0.07	-0.02
	Non Family	-0.22	0.27	-0.69	-0.49	-0.40
	Offspring	-0.45	0.03	0.54	0.52	0.47
Multiple support	Multiple	1.49	-1.24	0.34	-0.72	-0.33
	single	-0.28	0.23	-0.06	0.13	0.06
Frequency of support	Often	0.43	-0.68	-0.29	0.35	0.23
	Rarely	-0.26	0.42	0.18	-0.21	-0.14
Type of support	Household help	-0.28	-0.40	0.05	-0.06	-0.02
	No Household help	1.05	1.53	-0.18	0.23	0.06
	Paperwork/Pers. Care	1.22	0.34	0.05	-0.18	-0.11
	No Paperwork/Pers. Care	-0.68	-0.19	-0.03	0.10	0.06
Welfare regime	De-familialisation	-0.35	0.15	1.03	-0.48	-0.56
	Towards de-familialisation	0.13	-0.23	-0.67	1.11	-1.04
	Familisation	0.76	0.29	0.46	0.74	1.19
	Other	-0.10	-0.12	-0.77	-0.77	0.87

Table 3: Coordinates of variable categories for values displayed in Figure 6. Columns Dim 1 and Dim 2 were used as the X and Y axis in Figure 6.

Dimension	Var. Explained	Cum. Var. Explained
dim 1	23.46	23.46
dim 2	15.24	38.70
dim 3	11.80	50.50
dim 4	11.30	61.80
dim 5	11.02	72.82
dim 6	10.47	83.29
dim 7	8.59	91.88
dim 8	8.00	99.87
dim 9	0.13	100.00

Table 4: Variability explained by the dimensions extracted in MCA (%) reported in Figure 6.

Variable	Category	Dim 1	Dim 2	Dim 3	Dim 4	Dim 5
Type of alter	Family	1.57	-1.04	0.85	0.74	-0.40
	Non Family	1.04	1.32	-0.34	0.06	0.64
	Offspring	-0.57	-0.19	-0.05	-0.15	-0.11
Multiple support	Multiple	1.21	-1.38	-0.60	-1.31	-0.49
	Single	-0.20	0.23	0.10	0.22	0.08
Frequency of support	Often	-0.04	-0.55	-0.66	0.28	0.62
	Rarely	0.03	0.45	0.54	-0.23	-0.50
Type of support	Personal care	1.66	-1.73	0.23	2.03	-0.83
	No Personal care	-0.11	0.12	-0.02	-0.14	0.06
	Household help	0.94	0.29	-0.61	-0.24	-0.36
	No Household help	-0.58	-0.18	0.37	0.15	0.22
	Paperwork	1.43	-0.69	1.29	-1.02	1.26
	No Paperwork	-0.21	0.10	-0.19	0.15	-0.18
	Childcare	-0.73	-0.27	-0.04	-0.17	-0.07
	No Childcare	1.05	0.39	0.06	0.25	0.10

Table 5: Coordinates of variable categories for values displayed in Figure 7. Columns Dim 1 and Dim 2 were used as the X and Y axis in Figure 7.

Dimension	Var. Explained	Cum. Var. Explained
dim 1	34.74	34.74
dim 2	18.65	53.39
dim 3	12.46	65.85
dim 4	11.82	77.67
dim 5	10.26	87.93
dim 6	9.09	97.02
dim 7	2.72	99.74
dim 8	0.26	100.00

Table 6: Variability explained by the dimensions extracted in MCA (%) reported in Figure 7.

Variable	Category	Dim 1	Dim 2	Dim 3	Dim 4	Dim 5
Type of alter	Family	1.50	-1.14	0.14	0.77	0.50
	Non Family	1.07	1.13	-0.67	0.05	-0.03
	Offspring	-0.57	-0.12	0.16	-0.15	-0.08
Multiple support	Multiple	1.19	-1.21	1.06	-0.95	-0.59
	Single	-0.20	0.20	-0.18	0.16	0.10
Multiple support	Often	-0.08	-0.62	-0.44	-0.29	-0.29
	Rarely	0.06	0.50	0.36	0.23	0.23
Type of support	Personal care	1.57	-1.91	-0.55	0.14	1.07
	No Personal care	-0.11	0.13	0.04	-0.01	-0.07
	Household help	0.96	0.27	-0.00	-0.45	-0.22
	No Household help	-0.59	-0.17	0.00	0.28	0.14
	Paperwork	1.40	-0.71	0.67	0.85	-0.07
	No Paperwork	-0.20	0.10	-0.10	-0.12	0.01
	Childcare	-0.73	-0.19	0.20	-0.15	-0.09
	No Childcare	1.05	0.28	-0.29	0.21	0.14
Welfare regime	De-familialisation	0.16	0.48	1.14	-0.25	0.59
	Towards de-familialisation	0.03	-0.00	0.01	0.94	-1.42
	Familisation	-0.50	-0.66	-0.60	0.27	0.77
	Other	0.31	0.14	-0.67	-0.94	-0.04

Table 7: Coordinates of variable categories for values displayed in Figure 8. Columns Dim 1 and Dim 2 were used as the X and Y axis in Figure 8.

Dimension	Var. Explained	Cum. Var. Explained
dim 1	25.82	25.82
dim 2	14.22	40.05
dim 3	10.37	50.42
dim 4	9.35	59.77
dim 5	9.20	68.97
dim 6	8.41	77.38
dim 7	7.80	85.17
dim 8	6.57	91.74
dim 9	6.10	97.84
dim 10	1.97	99.81
dim 11	0.19	100.00

Table 8: Variability explained by the dimensions extracted in MCA (%) reported in Figure 8.
